# Familial Influences on Adolescent E-Cigarette and Cannabis Use: A Qualitative Analysis

**DOI:** 10.1080/29973368.2026.2732043

**Published:** 2026-09-21

**Authors:** Elizabeth T. Couch, Kayla Lieuw, Claudia Guerra Castillo, Bonnie Halpern-Felsher, Benjamin W. Chaffee, Kristin S. Hoeft

**Affiliations:** a University of California San Francisco, San Francisco, CA, USA;; b Stanford University, Stanford, CA, USA

**Keywords:** adolescents, cannabis, e-cigarettes, family, qualitative, tobacco, tobacco prevention

## Abstract

Family factors influencing adolescent cigarette smoking are well described, but specific family influences of e-cigarettes and cannabis behaviors are underexamined. The objective of this study was to explore the role of family in adolescent e-cigarette, other tobacco, and cannabis use. Semi-structured interviews with 47 California adolescents, aged 13–17, who had ever used tobacco products were conducted between May 2020 and December 2021. Thematic analysis techniques identified unifying themes related to family factors and adolescent e-cigarette and cannabis use behaviors. Family members often played a role in initiating or facilitating access to substances. Some participants perceived that their parents displayed permissive attitudes toward e-cigarettes and cannabis, often setting weaker boundaries and allowing exceptions to otherwise stricter rules for combustible tobacco. Others expressed fear of being caught using tobacco by their family and used more easily concealed products (e.g., small, odorless devices) and other strategies to hide use from parents and family. For some, experiencing parental punishment or observing addiction in the family only partly deterred e-cigarette and cannabis use. Future research and education initiatives should focus on developing strategies that reduce youth access to e-cigarettes and cannabis by addressing and leveraging the nuanced role family plays in adolescent substance use behaviors.

## Introduction

The prevalence of e-cigarette and cannabis use among adolescents in the United States is a major public health concern. E-cigarettes continue to be the leading tobacco product used among adolescents, with 2.8 million middle and high school students reporting current e-cigarette use in 2023 (Birdsey, 2023). Cannabis use is also common among youth, with 30.7% of 12th graders reporting past-year cannabis use in 2022 (Miech et al., 2023). Co-use of cannabis and tobacco is also prevalent among adolescents (Nguyen et al., 2019), with some studies finding co-use to be more common than use of each product alone (Liu et al., 2023; Schauer & Peters, 2018). Use of these products by adolescents has potential short- and long-term health implications. E-cigarettes are associated with adverse respiratory outcomes (Chaffee et al., 2021; Hamberger & Halpern-Felsher, 2020) and contain nicotine, which can harm adolescent brain development and increase the risk of addiction (Taylor et al., 2014). Similarly, cannabis use among adolescents is associated with asthma (Silverman et al., 2024), cognitive impairment, mental health issues (psychosis, major depression, and suicidal ideation), decreased academic performance, and substance use addiction (National Academies of Sciences, Engineering, and Medicine, 2017; Sultan et al., 2023). Understanding factors that influence experimentation and use of these products is essential in preventing and mitigating their use among adolescents.

Findings from prior studies have shown how family factors influence a number of adolescent risk behaviors, including substance use (Madras et al., 2019). For example, adolescents with parents who smoke cigarettes are more likely to initiate smoking themselves (Amutah-Onukagha et al., 2023; Otten et al., 2007; Peterson et al., 2006; Vuolo & Staff, 2013). Sibling use of cigarettes and other substances has also been shown to predict use among adolescents (Amutah-Onukagha et al., 2023; Bahr et al., 2005; Otten et al., 2007; Peterson et al., 2006; Vuolo & Staff, 2013). In contrast, children from families who enact strict household rules about tobacco and other substances are less likely to use than children without such rules (Proescholdbell et al., 2000; Staff et al., 2024; Wu & Chaffee, 2020). Moreover, supportive familial relationships that foster cohesion and closeness have been shown to reduce the likelihood of adolescents’ engagement in substance use (Amutah-Onukagha et al., 2023; Padilla-Walker et al., 2008), emphasizing the important role family plays in determining risk behaviors.

Although past research highlights the central role of family in both promoting and preventing adolescent substance use behaviors, fewer studies have explored the nuanced connections between family factors and adolescent e-cigarette and cannabis use. Each of these products are now used by adolescents far more commonly than conventional cigarette smoking. Parents and adolescents may hold very different attitudes toward e-cigarette and cannabis use due to several factors, including the medicalization and legalization of cannabis, perceptions of reduced harm of e-cigarettes compared to other tobacco products, and the cultural normalization of such products. Existing research indicates that family tobacco use and secondhand smoke exposure predict adolescent e-cigarette use (Adebisi et al., 2025; Wang et al., 2019). Moreover, parental attitudes, especially those that are more accepting of e-cigarettes and cannabis, may contribute to positive perceptions and increased use among adolescents (Fite et al., 2018). Similarly, family dynamics, communication, and attitudes play a role in shaping adolescent attitudes and behaviors related to cannabis use. Some studies suggest that parental monitoring, communication, and attitudes can either promote or deter youth from experimenting with cannabis (Cardenas et al., 2022), whereas others have shown that clear parental boundaries and open conversations about risks aid in preventing or reducing cannabis use among youth (Napper et al., 2016).

Qualitative research can provide insight into the underlying dynamics and decision-making that influence adolescent substance use within families, potentially informing future public communication and prevention programs. However, qualitative studies investigating the role of family in shaping youth use of e-cigarettes and cannabis are limited. In one study of regular e-cigarette users, most adolescents reported having a family member who also used e-cigarettes, and they perceived neutral or positive attitudes from parents regarding their own e-cigarette use (Alexander et al., 2019). A study of Australian parents found that perceptions of e-cigarettes were multifaceted; many parents viewed them as less concerning than other adolescent issues and believed adolescent experimentation was inevitable (Rose et al., 2025). Another study with Native Hawaiian and Pacific Islander youth found that parents and family members were permissive of youth e-cigarette use, often normalizing its use through their own behaviors (Marshall et al., 2024). Other studies have demonstrated implicit approval of e-cigarette use by parents for cessation among adolescents who smoke cigarettes (Alexander et al., 2019; Hammal & Finegan, 2016; Kirkcaldy et al., 2019). Additional research is warranted to examine how adolescents explain their own decisions related to tobacco and cannabis use, especially in the context of expanded legal access to cannabis and in light of marketing and public discourse that often present e-cigarettes as a less harmful alternative to combustible cigarettes.

In this study, we examine the role that family (i.e., parents, siblings, and other relatives) have on adolescent e-cigarette, other tobacco (i.e., cigarettes, cigars, etc.), and cannabis use behaviors. Specifically, this study aimed to examine how parents, siblings, and extended family influence adolescents’ decisions and behaviors regarding e-cigarettes and cannabis. Although e-cigarettes and cannabis differ in their legality, perceived risk, and use context, they share overlapping access pathways, use patterns, and shared family-level influences, warranting joint examination. Through qualitative interviews, we explore detailed, firsthand experiences from adolescents about the role that family plays in encouraging or preventing their substance use. Their stories enhance our understanding of the intricate parental and familial elements impacting their experimentation and use of these products. Such information can guide tobacco control policies and shape public health communication strategies for adolescents and their families.

## Methods

### Participation and recruitment

We conducted a qualitative, cross-sectional study using semi-structured interviews. In total, 47 adolescents participated (mean age 15.4 years; 66% female; 68% rural). Participants were recruited from two samples. The first sample was recruited between May and June 2020 from an existing cohort study of students 13–17 years old at eight rural high schools in California. In 2019–2020, 9th and 10th graders at those schools were enrolled in a prospective cohort study, which included a survey (Chaffee et al., 2021). Those adolescents who self-reported tobacco or nicotine product use in the past 30 days had parental permission to be contacted again for research, and themselves had agreed to be contacted for other research opportunities, were invited by email or text message to participate in the current study.

To broaden geographic and demographic diversity, a second sample of participants was recruited using ads on social media (Facebook and Instagram) targeting parents of California adolescents between the ages of 14–17. From September 2020 to February 2021, we sought adolescents throughout California, including urban, suburban, and rural areas, who reported using e-cigarettes or other tobacco products (e.g., cigarettes, cigars) at least 3 times in their life. Data from these two samples also contributed to two prior publications (Chaffee et al., 2025; Guerra Castillo et al., 2024). Those studies focused on nicotine addiction and counterfeit vaping devices, whereas the present analysis uniquely examines family influences. Although participants overlapped, this manuscript addresses a distinct research question with separate coding and thematic analysis. Racial and ethnic composition was not consistently collected across participants and therefore could not be systematically examined.

Adolescent assent and parental consent to participate were collected for all study participants. Participants were provided with a gift card to a major online retailer valued at $20 for the first sample and $40 for the second sample. The incentive amount rose in the second sample to improve recruitment. All study procedures were approved by the University of California San Francisco Institutional Review Board.

### Interviews

Semi-structured interviews were conducted remotely by videoconference. All interviews were conducted by one of two experienced interviewers (KSH and CGC) and lasted 45 to 60 minutes. The interviews were conducted using a semi-structured guide aimed at addressing several tobacco-related topics, including participants’ personal experiences related to tobacco and cannabis use and their perceived access to products, particularly in the context of the COVID-19 pandemic. Discussions about the role of family in tobacco, nicotine, and cannabis-related behaviors were not directly prompted; instead, they were revealed by participants during the interview process. This topic was a strong and universal emergent theme, and comprises the focus of this paper. Further details about the interview guide can be found in Guerra Castillo et al. (2024) and Chaffee et al. (2025), which describe the development and use of semi-structured prompts and provide additional examples.

### Data analysis

Interviews were audio-recorded, transcribed verbatim and de-identified. Transcripts were imported into the Dedoose Web Application for data analysis (Dedoose, 2022). A codebook was developed based on the interview guide (e.g., “vaping”, “cannabis”, “tobacco access”) and emergent topics (e.g., “parents,” “controlling parents,” “siblings”, “extended family,” “getting caught”, “punishment”) using the approach of Braun and Clarke (2006). Two researchers (KL and CGC) independently read through and coded the transcripts in an iterative process. Intercoder reliability was assessed by dual coding 5 transcripts (~10%) and checking coded overlap and accuracy by KSH; any discrepancies were resolved through discussion, and adjustments were made to code definitions and recoded. The excerpts were then analyzed to identify the themes across participants. Data from both recruitment samples were analyzed together, with attention to identifying patterns that were consistent across participants rather than sample-specific differences.

## Results

Forty-seven in-depth interviews were completed, with 25 participants in the first sample and 22 participants in the second. Participant mean age was 15.4 years, with the majority identifying as female (66%). Over half of participants (68%) resided in rural areas and had two parents in their household (67%). Nearly all participants (98%) reported ever using e-cigarettes in their lifetime, and 66% reported use of e-cigarettes in the past 30 days. Although cannabis use was discussed by some participants, eligibility was based on e-cigarette use and systematic measures of lifetime or past 30-day cannabis use were not collected.

Participants discussed the direct and indirect ways that their families, including siblings, cousins, uncles, aunts, parents, and grandparents, influenced their beliefs and behaviors around e-cigarettes, other tobacco products, and cannabis. The extent of family use varied across participants and played an important role in adolescent decision-making around e-cigarette and cannabis use. Despite all participants having a history of tobacco and/or nicotine use, the products and patterns of use were notably influenced by family, particularly parental influence. Three main themes emerged from the analyses: (I) Family use promoting access, (II) Perceptions of parent permissiveness (with stipulations), and (III) Parental punishment and other consequences. A summary of the themes and sub-themes, along with their conceptual contributions, is presented in Table 1.

### Theme I. Family use promoting access

For many youth participants, their first experience with e-cigarettes, other tobacco, or cannabis was around family. Many participants reported initiating use of e-cigarettes and cannabis with family members who use. When asked to describe their initiation experience, older siblings were often involved or present at the time of initiation.

“I was 13. I was actually with… my oldest sister and her friend and they’re doing it [cannabis] and I wanted to try it and I did.”

– Participant 30, Female, age 15

“I think it was summer of my freshman year, I went to see some family, and one of them had… a nicotine one [e-cigarettes], and I hadn’t tried nicotine before.”

– Participant 31, Female, age 17

“My brother, me and him were fishing and then he just said, ‘Hey, you wanna try it [smokeless tobacco]?’, and I was, ‘Sure’”

– Participant 8, Male, age 16

Whereas initiation of cannabis was usually framed as a spontaneous decision, participants often recounted instances in which their older siblings facilitated their access to cannabis after initiation. Participants seemed to encounter minimal difficulty in obtaining cannabis products from their siblings and even discussed situations in which they used cannabis together.

Interviewer: How were you getting the weed [cannabis]?

Adolescent: My half-sister, step-sister, she was a manager at a dispensary… so, I got (laughs) quite a bit of gifts from her.

– Participant 35, Male, age 17

“Whenever I do wanna use cannabis, I have access to it because my sister usually has it….every once in a while, I’ll just be like, ‘Oh, yeah. I’ll use it [cannabis].’ Because my sister likes it when I use it ‘cause she thinks I’m… Well, I’m always really funny to her when she’s really high. She always laughs at everything I say (laughs).”

– Participant 23, Female, age 14

Extended family members, such as uncles and cousins, also emerged as a source of access to these products. Though some participants acknowledged older family members purchasing products for them, many participants who did not own their own e-cigarettes discussed borrowing or sharing their family member’s products during holidays and other gatherings.

Interviewer: How were you getting your nicotine salts and everything from before?

Adolescent: Um, mainly like the mini mart…or I’d have somebody to get it for me…actually my uncle.

– Participant 16, Female, age 15

“…last time during Thanksgiving, I think we went down to our cousin’s house and my cousin, she’s around my age, she had one [e-cigarette] and she uses it quite a lot. …So, she would pass me it every once in a while and I would just blow….”

– Participant 42, Female, age 14

It was uncommon for parents to purchase e-cigarettes, other tobacco, or cannabis products for their adolescent children. However, in a few cases, parents who used products unknowingly facilitated access by leaving products accessible in the house. Participants reflected on how the presence of products in the house normalized and encouraged their experimentation and use. For example, one participant recounts using her mom’s Puff Bar, a disposable e-cigarette that comes in a variety of flavors.

“It [e-cigarette] was just around a lot more. I had access to it…much easier. So…I would grab it…. Actually, it was because it was around so much that I did it more. …Um, yeah, I just grabbed my mom’s [Puff Bar].”

– Participant 33, Female, age 14

In families in which e-cigarette use was normalized, in some instances, more than one family member provided information and access to the newest products.

Adolescent: I learned about it [new e-cigarette brand] because my, everybody in my family vapes and they just had them. So, um, I went and got one.

Interviewer: And when you say everybody in your family, like who’s in your family?

Adolescent: So, um, I have my mom, my sister and my brother and, um, they vape, or and my sister’s fiancé.

Interviewer: And so did you try theirs first or did you hear about it and went out and bought your own?

Adolescent: I tried theirs first.

– Participant 16, Female, age 17

“If he [my brother] gets something new, he’ll share with me, if I get something new, I’ll share with him, and so on and so forth. But he was definitely the one that first kind of introduced it [e-cigarettes] to me.”

– Participant 15, Male, age 15

Overall, family members served not only as role models but also as direct facilitators of access, shaping both initiation and continued use among adolescent participants.

### Theme II. Perceptions of parent permissiveness (with stipulations)

Despite a general awareness that parents would prefer that their children not use tobacco, nicotine, or cannabis products, some youth perceived parents to be relatively permissive of their e-cigarette and cannabis use, only setting minor stipulations or boundaries. For example, parents were perceived to be more lenient toward e-cigarette and cannabis use as adolescents approached adulthood, with one participant expecting that her mom would likely purchase e-cigarettes for her once she turned 18, noting that as long as she was 18 years old, her mom would be fine with it.

“I’d probably just have my mom take me to get it [e-cigarette], you know? ‘Cause you can go and you can’t buy technically buy weed or stuff like that until you’re 21, but most people will just have their friends or their parents go with them so they can buy it for them…I mean it’s not like I’m gonna have to be having her pay for it, you know?”

– Participant 29, Female, age 16

Other participants acknowledged their parents’ leniency toward tobacco, nicotine, and cannabis use by older adolescents in and around their homes. Some parents seemed to permit use among older adolescents as long as they were being discrete. Factors such as visibility to younger siblings and concern about how they were perceived externally indicated some of the limits of parental permissiveness.

“He’s [my boyfriend] two years older than me. So, I don’t know. I think my mom gives him just a little bit more of a leeway. ‘Cause I know she doesn’t want me to be into vaping and stuff.”

– Participant 7, Female, age 17

“So, my mom would just really be like, ‘Oh, just go do it in the backyard.’ When my sister would come over and she would go smoke [cannabis] outside, my mom wouldn’t want her to smoke up front [in front of the house] because, I guess, she wouldn’t wanna put a bad image for herself out there. Oh, those people let their kids smoke and stuff like that, so. But my sister’s 18, 19 now.”

– Participant 29, Female, age 16

In some cases, adolescents believed that their parents were aware of their cannabis and e-cigarette use but chose not to address it. Parents did not appear to provide clear boundaries or guidance about substance use in and out of the home.

“I haven’t really talked to them [my parents] about it [smoking cannabis], but they know I’m gonna smoke.”

– Participant 11, Male, age 15

Some participants also mentioned that their parents became more lenient about their use over time, attributing such leniency to the myriad of challenges adolescents were facing in their lives. Examples raised included parent separation, parent illness (cancer), loss of a parent or loved one, and external stressors like the COVID 19 pandemic and school closures. Adolescents seemed to interpret their parents’ tolerance as an unspoken recognition of the use of these products as a coping mechanism amid the complexities of their personal and social environments, creating a situation in which e-cigarettes and cannabis were more accepted.

“I never really used it frequently, but the end of my sophomore year my dad passed away. And so after that I started smoking [cannabis] like more regularly…she [mom] never really got mad at me.”

– Participant 7, Female, age 17

“He [dad] just didn’t really wanna know about it [cannabis e-cigarette use] if I used it before quarantine [from COVID-19]. But now, he doesn’t really care…I’m pretty sure he’s known when he was giving me and my friend food that we were high.”

– Participant 23, Female, age 14

In some cases, adolescents reported instances in which their parents provided cannabis products to their children for medicinal or therapeutic purposes. Adolescents felt that parents reluctantly tolerated cannabis use for their children who experienced mental health struggles, such as anxiety and depression. Cannabis was viewed as a coping mechanism or lesser evil in the face of significant emotional challenges. This pattern appeared more commonly in relation to cannabis than e-cigarettes, particularly when adolescents described parents framing cannabis use as therapeutic, protective, or preferable to other risks.

“I know that one of my friends who always has a dab pen [for smoking cannabis], her mom started just buying it for her, just starting buying carts [cartridges] for her because she also has…the whole mental health thing. She knows that…her daughter’s way more stable when she has something that she can hit. So, she buys her the carts ‘cause she doesn’t want her to be really, really, really sad all the time.”

– Participant 23, Female, age 14

“…My dad just buys it [cannabis] for her [older sister] from dispensaries or from his friends…I think that it’s okay because she’s about to be 18…She wants to get a medical card because she does need it for her mental health…I don’t really care that much if my dad buys it for her…I’m not like, ‘Oh, my God. Dad, don’t buy that for her. She’s underaged.’ It makes her happy. And she’s not gonna have panic attacks. So, I’m fine with it. As long as she’s happy.”

– Participant 23, Female, age 14

Participants also described their parents’ reluctant acceptance of their cannabis and e-cigarette use as long as they were being “safe.” Participants perceived their parents to believe that using these products within the confines of their home was considered a safer option compared to using them outside or “on the streets.” Although their parents likely prefer that they don’t use the products at all, they seemed more amenable to the idea of their children doing so within the home, where they could ensure the safety of the product, as well as their adolescent’s physical safety.

“I guess, as long as she [mom] knew I would be responsible with it [cannabis]. And she kind of was just like the parent who said that they’d rather me be doing something like that…in the safety of like my own house, not out on the streets or something. So, she makes sure everything is safe….”

– Participant 7, Female, age 17

“I mean, she [mom] was okay knowing that I did it [cannabis], she just wanted to make sure that I was being safe about it. I don’t think she’d be happy that I’m doing it, but…I don’t think she’d be as mean about me doing it. She just wants to make sure I’m doing it while I’m being safe.”

– Participant 44, Female, age 17

Participants with permissive or ambivalent parents were often able to use products around their parents’ preferences, such as using outdoors or at home. These participants rarely felt a need to reduce or avoid use, but rather could use within the parameters of their parents’ preferences and avoid conflict. Overall, this dynamic suggests a nuanced approach, where parental attitudes set boundaries that, while not fully endorsing certain behaviors, may imply a level of tolerance in specific situations.

### Theme III. Parental punishment and other consequences

In contrast to the perceived permissiveness or ambiguous parental attitudes toward the use of certain substances, some adolescents reported their parents being more prohibitive toward their use of e-cigarettes, other tobacco, and cannabis. Such prohibitive views and disapproval seemed to influence adolescents’ substance use behaviors in complex ways. For many participants, parental punishment or fear of disappointing parents prompted concealment of use, while for others, these same prohibitive attitudes, and witnessing negative consequences of substance use within the family, served as motivation to avoid use altogether or to quit.

Concealment of use seemed to be motivated by fear of punishment or disappointing parents and family members. The level at which adolescents concealed their e-cigarette and cannabis use from their parents and family seemed to vary across participants. Some adolescents recognized that although their parents were aware of their use, they did not reveal the full extent of their behaviors.

Adolescent: I was really bad. …I would be smoking [cannabis] like all night and then I’d wake up at 6:30 a.m. and then smoke before I went to school. And then my friends would let me use their stuff at school after like every class.

Interviewer: Okay…does she [mom] know you were doing it before?

Adolescent: No. She thinks I only did it like once (laughs).

– Participant 9, Female age 15

Many participants expressed concern about getting caught at home with an e-cigarette in their possession, suggesting that some parents had drawn clear boundaries about using products. Some adolescents were so worried that they would avoid bringing their products into their homes and hide them elsewhere.

“It [cannabis vape] was my own and I didn’t want to keep it in the house ‘cause I was scared of being caught. So, I still had my own, but that didn’t mean I used it more often because I was scared to be caught in the house with red eyes or something like that. People talk, they had stories about getting caught all the time.”

– Participant 34, Male, age 16

“Well, before COVID I was going out [to skateboard] every single day. Part of the reason was because, when I was vaping [e-cigarettes], I wouldn’t bring it into my house ‘cause I was afraid I’d get [patted] down or something. ‘Cause I’ve already been caught three times before then. So, if I knew, if I got caught again, I was screwed and a way to avoid getting caught was just not bringing [the device] in the house… …I was too afraid to bring it inside. (laughs)”

– Participant 39, Male, age 16

When adolescents did bring products into their homes, they would find discreet places to hide them, such as a jacket pocket or a specific area in a closet.

“I just, uh, put it [cannabis] in one of my jacket pockets in my closet, yeah.”

– Participant 20, Male, age 15

“I have a pocket in my jacket, I kept it [e-cigarette] in my…because there was my jacket hanging up in my closet. I’ll keep it in there, in the pocket.”

– Participant 38, Male, age 13

“But I used to keep it [cannabis] in a pretty safe space, up in my…in the corner of my closet, and I never really had to check if it was there ‘cause my parents don’t really search my possessions, or anything, so…they haven’t really been a concern for me.”

– Participant 5, Male, age 15

Certain characteristics of e-cigarettes made them relatively easy to hide, such as their lack of smell, small size, and similarity to USB devices. Similarly, edible cannabis products were viewed as being easier to conceal due to their lack of smell. The ability to hide these products from their parents and other authority figures, like teachers, was a key component behind product choice.

“Sometimes, we would do it [vape cannabis] when we would come to my house if my friend had a dab pen because I wouldn’t use my sister’s stuff because I didn’t want it to smell.”

– Participant 23, Female, age 14

“…we would [use edible cannabis], because this smoking thing wouldn’t work out, especially at school, and I wouldn’t do it at my house, because then my parents would know. We started getting edibles from this other kid at school, and they’re little chocolate candies. It was easier to hide, because, you know, you couldn’t see the smoke or anything.”

– Participant 31, Female, age 17

Some adolescents colluded with friends to try to hide vaping products from their parents. Often, the friends were neighbors because the close proximity allowed them to sneak products without getting caught.

“I was grounded for very long time and I cut a hole in my window screen. So, I would ask my friend who was right next door, and he would come. And then at night he would come slip it [e-cigarettes] through the window and let me use it. Then I would slip it back through the window and we’d do that for a little while I was grounded.”

– Participant 39, Male, age 16

Interviewer: How do you get your vaping products [e-cigarettes] during the shelter-in-place?

Adolescent: My friend would throw them over the fence [to me].

– Participant 6, Female, age 17

In some cases, parent punishment or observed family consequences drove adolescents to quitting or avoiding use. Some adolescents who had been caught and punished by parents in the past were deterred from using again. The fear of getting caught or being caught acted as a wake-up call for them to quit or consider quitting.

“Yeah, but my parents obviously found out, so, (laughs) yeah. That’s maybe what did traumatize me. But it wasn’t, not traumatized, but pushed me away from doing it [cannabis vaping] over again. Just ‘cause I didn’t want to get caught again.”

– Participant 34, Male, age 16

“I guess she [mom] came to say good night or check on me and I wasn’t in my room. And then, my dad came out on the porch and smelled it [cannabis joint]…. They’re not really anti-pot, they’re just anti-underage pot. So, she just took it…. and she basically just said that I couldn’t get my learner’s permit until I’m 16. …I don’t think it’s worth the punishment that would be inflicted if it was found out.”

– Participant 20, Male, age 15

For those who continued to use e-cigarettes and cannabis, many expressed feeling some level of guilt about their actions. Many adolescents worried that their use of e-cigarettes and cannabis could jeopardize a positive relationship with their parents, leading some to second guess their decision to use.

“I just feel guilty [about using cannabis] because my mom caught me and that just makes me feel bad to like do stuff like that behind her back.”

– Participant 9, Female, age 15

Adolescent: I just hid it in my pocket. And we [mom and I] were just talking for a little bit and then she [mom] walked out, and after that, I just thought to myself, what am I doing? This is stupid. You shouldn’t be scared for your mom to walk in in the middle of the night, you shouldn’t be doing anything bad at the middle of the night when your mom walks in, and you shouldn’t be hiding something in your pocket because you don’t… because your mom is somebody you love and you don’t want to lose the trust of somebody you love.

Interviewer: What do you think she would think or do if she knew that you were vaping?

Adolescent: Just like lose trust in me, and she would still love me the same, but just lose trust in me, not have that same bond you had before, and that’s something I don’t want to lose.

– Participant 38, Male, age 13

Adolescents’ feelings of guilt and responsibility related to their use of these products extended beyond their parents to include younger siblings and other family members. Some participants concealed their use, expressing a sense of responsibility for shielding their younger siblings from seeing them using or being under the influence of nicotine or cannabis. In specific instances, the presence of younger siblings or other family members appeared to discourage participants from using products altogether, particularly when that person was at home. Furthermore, for a few participants, younger siblings acted as a powerful motivator to continue remaining abstinent after quitting.

“Because…my little sister’s around a lot. And I wanna make sure that I can actually function properly (laughs) because I can get high really, really, really, really easily. … So, I wanna make sure that I can function normally most of the time so that I can help my little sister. So, I don’t do it [vape cannabis] unless my little sister’s at her mom’s house.”

– Participant 23, Female, age 14

“So, cutting down or cutting off [using e-cigarettes] in general is good and it’s not what I want my godsister to see…. It’s been going good…keep my head on straight because I don’t want to be failing as an example for her because she does look up to me.”

– Participant 26, Male, age 16

Family history of tobacco product use and addiction also appeared to influence adolescents’ decision-making related to use. Adolescents who had experienced seeing a family member struggle with addiction expressed apprehension and caution toward their tobacco and cannabis use. However, these concerns did not always deter youth from using the products. Although adolescents were aware of the risk of addiction, they also felt that they had some level of control over their use and could stop before they became addicted themselves.

“I mean, I definitely didn’t want to get addicted [to e-cigarettes] at all. I was always scared of that because I have grown up in, just with my whole family, not even my mom… it’s a lot of alcohol and drug [inaudible] but I knew that I wouldn’t do it to the point to get addicted.”

– Participant 33, Female, age 14

“[explains that father is addicted to cannabis…] It’s just, honestly, it’s mainly just the cost. Honestly, if I think if I start smoking weed, I’d probably get addicted too. I’m really not trying to go into debt just because of some weed.”

– Participant 29, Female, age 16

Overall, these findings suggest that parental disapproval and family history exert influence through both emotional mechanisms, such as guilt and fear of disappointment, and behavioral mechanisms, including concealment and cessation, underscoring the complex ways in which familial norms shape adolescent decisions about substance use.

## Discussion

We utilized in-depth qualitative methods to examine familial influences of adolescent e-cigarette, other tobacco, and cannabis use. Participants’ families fostered diverse expectations and environments regarding vaping and cannabis, influencing adolescents’ use intentions and behaviors. Though all participants in this study had experience at some point using tobacco and nicotine products, their intentions to use and use frequency seemed to be influenced, in part, by their family’s attitudes and behaviors. Findings extend prior work by showing not only that family influence matters, but how it operates through conditional, relational, and product-specific pathways. The contribution of this study lies in identifying the ways adolescents interpret family behaviors and expectations, including permissiveness with boundaries, safety-oriented parenting and emotional responsibility within the family system.

Although peers are often noted as a primary influence of youth tobacco and other substance use, family members also play a pivotal and often overlooked role. For adolescents in this study, family members were often present during initiation and use of e-cigarettes and cannabis. Similar to previous studies, we found that adolescents with siblings and other family members who use e-cigarettes and cannabis have increased opportunities to use themselves (Fite et al., 2018; Vuolo & Staff, 2013). One study found that adolescents often have family members in their home who use e-cigarettes, and those devices were often the first products used by participants (Alexander et al., 2019). In our study, we found that participant’s older siblings and other family members were often involved in both initiation and intermittent access to these products, especially cannabis. We also found that parents and siblings played an important role in providing information about and sometimes accessing e-cigarette products. These findings align with recent evidence showing that parents express concern about adolescent vaping (Rose et al., 2025) yet may also struggle with role-modeling and inadvertently allowing access when they themselves use e-cigarettes (Marshall et al., 2024). Family appears to be one of several potential sources of access to these products. Studies have found several other potential factors aiding youth access to e-cigarettes. A study of high school students in California found that students are more likely to access products from friends rather than asking family members or buying products themselves (Meyers et al., 2017). Another study found that adolescents in Southern California often access tobacco products through peers at school, online, and at in-person retailers that do not consistently enforce age restrictions (Schiff et al., 2021). Understanding the various strategies that underage adolescents utilize to gain access to nicotine and cannabis products is imperative for enhancing enforcement strategies and reducing the availability of these products.

Many adolescents in this study perceived their parents to be somewhat permissive of their use of e-cigarettes and cannabis. In a qualitative study of youth in North Carolina and Florida, adolescent participants perceived their parents to be neutral or accepting of their e-cigarette use, especially when compared to cigarettes or other substances (Alexander et al., 2019). The impression that parents weighed e-cigarette use against other substances and situations that they perceived to be more harmful came out in the current study. Participants believed that their parents, though not entirely condoning their use, would rather they use cannabis and e-cigarettes in the home where they can be monitored more closely. Additionally, it was perceived that parents were more lenient toward adolescents’ e-cigarette and cannabis use in light of other, more demanding challenges adolescents may face. Cannabis, in particular, was sometimes viewed as therapeutic for adolescents facing stress, anxiety, or mental health challenges. Together, the idea that certain products or environments are viewed as safe, acceptable, or even beneficial under certain conditions was interpreted as positive messaging to youth about these products that encouraged their use.

Differences between cannabis and e-cigarette use also emerged. Cannabis was more often framed by adolescents as therapeutic or justified by parents in response to stress or mental health concerns, whereas e-cigarette use was more often described in relation to availability, concealability, and normalized use among family members. These distinctions suggest that family messaging about perceived acceptability may vary by substance.

Indeed, instances of parents enabling cannabis use for therapeutic purposes placed cannabis in a different category from e-cigarettes and tobacco. In some cases, adolescents reported parents purchasing cannabis products for their children to alleviate mental health symptoms, such as anxiety and depression. Parents were often perceived to be providing cannabis as a caring way to improve their child’s health and overall quality of life. Cannabis-derived products have been shown to be effective in treating certain childhood medical conditions, such as epilepsy and chemotherapy-induced nausea (Gibbard et al., 2021; Ryan et al., 2020; Wong & Wilens, 2017). However, research examining the use of cannabis to treat anxiety and depressive disorders has found inadequate supporting evidence (Black et al., 2019). Likewise, studies of cannabis use for mood disorders show mixed results (Feingold et al., 2015; Hayatbakhsh et al., 2007). Furthermore, a recently published study raised the alarm on the mental health impact of high potency cannabis products, which are increasingly more common now (Rittiphairoj et al., 2025). There is a need both for more evidence on the mental health effects of cannabis use among adolescents and for enhanced resources and communication directed toward parents and families to allow them to make informed decisions regarding cannabis and their children’s health and well-being.

Another important insight from this study is the tension between harm-reduction parenting approaches and the potential normalization of substance use. Some parents appeared to permit use under specific conditions (e.g., at home, for safety, or for perceived therapeutic benefit), which adolescents interpreted as tacit approval. While such approaches may be intended to reduce harm, they may also blur boundaries and shape perceptions of acceptability. Given the qualitative nature of this study, these findings should be interpreted as exploratory but highlight an important area for future research.

Adolescents living in households with strict rules about tobacco and e-cigarette use are less likely to use those products (Buu et al., 2022; Wu & Chaffee, 2020). In this study, participants were more apprehensive about using e-cigarette and cannabis products when they perceived serious consequences, whether direct parental punishment or guilt from defying their parents’ expectations or betraying their trust. These findings align with studies demonstrating that household exposure and rules shape adolescent use, with risk factors such as parent’s personal use, parents perceived harm, and youth knowledge of an e-cigarette ban in their home predicting relaxation of household e-cigarette bans over time (Maggs et al., 2024). However, despite parent disapproval in the current study, many participants continued to use e-cigarettes and cannabis, often concealing their use from family. Consistent with other studies (King et al., 2020; Wu & Chaffee, 2020), participants reported that their parents were unaware of the extent of their use. This aligns with findings from a large-scale quantitative study in Australia that found that parents of teenagers who had used e-cigarettes were considerably worse at predicting their child’s e-cigarette use (70%) than parents of adolescents who had not used e-cigarettes (97%) (Egger et al., 2024). Especially for e-cigarettes and cannabis, characteristics of the products, such as their small design or lack of smell, made their use easy to conceal. Participants also reported concealing use by colluding with friends or hiding products outside of the home, suggesting that parent disapproval and strict household rules are insufficient to prevent all youth from using e-cigarettes and cannabis. However, clear anti-tobacco expectations from parents, without exceptions for different products or contexts, are among the strongest factors cited by adolescents who never use tobacco in their decision to abstain (Couch et al., 2017).

The patterns identified in this study are consistent with concepts from established theoretical frameworks of adolescent behavior. For example, Social Cognitive Theory emphasizes the role of modeling and reinforcement within social environments (Bandura, 1986), which in our findings often occurred through family members attitudes and behaviors around substance use. Similarly, Problem Behavior Theory (Jessor, 2001) highlights how family norms and expectations interact with individual motivation to influence adolescents’ engagement in risk behaviors. This theory helps explain why some adolescents in our study may respond to parent punishment by hiding their use, while others stopped altogether. Although this study focused on family influence, adolescents’ behaviors and decisions occur within broader ecological contexts that include peers, schools, and community environments. Parents and family members do not operate in isolation, but rather within networks shaped by peer norms, culture, and public health messaging, especially in states with tobacco control efforts. Future research could more explicitly integrate these theoretical perspectives to deepen our understanding of how family and other social factors interact to influence adolescents’ use of tobacco and cannabis products.

Findings also suggest potential considerations for prevention and parent-focused education efforts. Interventions may benefit from supporting parents in setting clear and consistent expectations about substance use while also addressing how safety-oriented messaging may be interpreted by adolescents. Public health campaigns could incorporate guidance on communication strategies, boundary-setting, and modeling behaviors that reduce ambiguity around substance use expectations.

Several limitations should be considered when interpreting these findings. The study sample may not represent adolescents in California, generally. Importantly, the study was also conducted during the COVID-19 pandemic, which may have influenced family dynamics, supervision, and access to substances. The sample was also limited to adolescents with experience using tobacco and nicotine products. Therefore, findings may not capture the perspectives of never-users, who may experience different familial influences on decisions not to initiate. This restriction limits the generalizability of our results, as they likely over-represent permissive or substance-using family contexts and underrepresent protective environments. Findings may also be influenced by social desirability bias, as participants may have under-reported or misrepresented their substance use behaviors or family dynamics. Additionally, systematic data on lifetime or past 30-day cannabis use was not collected, which limits our ability to identify differences among users of different products. Differences in recruitment methods between samples may also have influenced participant characteristics. Although our study encompassed adolescents residing in both rural and urban/suburban regions, we did not identify significant variations in their experiences by geography. In addition, we were unable to systematically examine variations by individual- or community-level characteristics, which may shape adolescents’ experiences of family influences; these represent important opportunities for future research. Despite limitations, findings from this study help build a foundation for future research and contribute meaningful depth to the literature.

## Conclusion

This study provides valuable insights into how families shape adolescent substance use behaviors in the context of rapidly changing social norms around e-cigarette and cannabis use. Family members, especially siblings and extended relatives, often provided initial exposure and access to e-cigarette and cannabis products. Parents communicated mixed messages, with permissiveness in some contexts and prohibitive attitudes in others, leading adolescents to either conceal use or interpret permissiveness as tacit approval. In addition, emotional dynamics, including guilt, loyalty, and responsibility toward younger siblings, played a significant role in shaping adolescent decisions. Together, these findings demonstrate that familial influence extends beyond parental rules, encompassing the broader family system and emotional environment. By highlighting the tensions between permissiveness, prohibition, and emotional responsibility, findings from this study add depth to the existing literature and suggest new directions for prevention and investigation. Public health interventions and programs should leverage these insights by providing parents and caregivers with tools to set consistent expectations, foster open dialogue, and model healthy behaviors. Future research could explore how these influences vary across demographic contexts and examine strategies to support families in reducing youth access to and normalization of e-cigarette and cannabis use.

## Figures and Tables

**Table 1.** Summary of themes and subthemes related to familial influences on adolescent e-cigarette and cannabis use.

Themes	Subthemes	Conceptual insight	Example
Family use promoting access	Siblings and other family members introduce use; access via relatives; shared use	Family as a facilitator of access and normalization	*“If he [my brother] gets something new, he’ll share with me, if I get something new, I’ll share with him, and so on and so forth. But he was definitely the one that first kind of introduced it [e-cigarettes] to me.” – Participant 15, Male, age 15*
Perceptions of parent permissiveness (with stipulations)	Conditional allowance; safety framing; therapeutic cannabis	Conditional permissiveness with implicit acceptance	*“I mean, she [mom] was okay knowing that I did it [cannabis], she just wanted to make sure that I was being safe about it…” – Participant 44, Female, age 17*
Parental punishment and other consequences	Fear of being caught; concealment; guilt; quitting	Emotional and behavioral response to disapproval	*“It [cannabis vape] was my own and I didn’t want to keep it in the house ‘cause I was scared of being caught” – Participant 34, Male, age 16*
